# P-235. Non-utility of Catheter Tip Cultures in the Diagnosis of Catheter-related Bloodstream Infections

**DOI:** 10.1093/ofid/ofae631.439

**Published:** 2025-01-29

**Authors:** Kirtana Jonnalagadda, Ayush Goel, Anuj Ajayababu, Sarita Mohapatra, Hitender Gautam, Bimal Kumar Das, Manish Soneja, Naveet Wig, Benu Dhawan

**Affiliations:** All India Institute of Medical Sciences, New Delhi, India, New Delhi, Delhi, India; All India Institute of Medical Sciences, New Delhi, India, New Delhi, Delhi, India; ALL INDIA INSTITUTE OF MEDICAL SCIENCES NEW DELHI INDIA, NEW DELHI, Delhi, India; All India Institute of Medical Sciences, New Delhi, New Delhi, Delhi, India; All India Institute of Medical Sciences, New Delhi, India, New Delhi, Delhi, India; All India Institute of Medical Sciences, New Delhi, New Delhi, Delhi, India; All India Institute Of Medical Sciences, Delhi, Delhi, India; All India Institute of Medical Sciences, DELHI, Delhi, India; All India Institute of Medical Sciences, New Delhi, India, New Delhi, Delhi, India

## Abstract

**Background:**

Catheter-related bloodstream infections (CRBSIs) affect approximately 3 to 27% of patients. As per IDSA (2009) criteria, concordant organisms from blood and catheter tip cultures, or specific criteria met by two blood samples are required for CRBSI diagnosis. Catheter tip cultures necessitate removal of the line and entail increased healthcare costs. Emerging evidence questions the reliability of this labor-intensive practice in diagnosing CRBSI. We aimed to determine the utility of catheter tip cultures in diagnosing CRBSI at our Institute.

Catheter tip culture and Blood culture results

**Figure:**
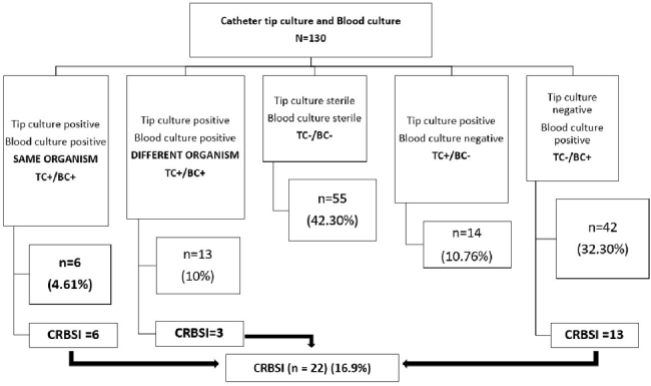

CRBSI: Catheter-related Bloodstream Infection; TC: Catheter tip culture; BC: Blood culture; + or - , growth or no growth

**Methods:**

We conducted a prospective observational study in patients with suspected CRBSI. Catheter tips and paired blood samples collected within 48 hours, were sent for culture. CRBSI was diagnosed as per IDSA 2009 guidelines. The utility of catheter tip cultures in diagnosing CRBSI was calculated.

**Results:**

One-hundred and thirty events of suspected CRBSI were included in 116 patients. Sixty-two percent of patients were male with a mean age of 43.75 ± 16.5 years. The prevalence of CRBSI was 16.9%, of which only 27% of CRBSI were identified by catheter tip cultures. The sensitivity, specificity, positive predictive value, and negative predictive value of catheter tip cultures in diagnosing CRBSI were 27.3% (10.7- 50.2), 77.8% (68.8-85.2), 20.0% (10.4- 35.0) and 84.0% (79.9-87.4) respectively. Catheter tip colonization was observed in 20.8% of catheter tip cultures. All repeat blood cultures sent in the following two weeks in patients with catheter-tip colonization, were sterile. Amongst patients with CRBSI, Multidrug-Resistant *Acinetobacter baumanii* and *Klebsiella pneumoniae* were the most frequently isolated organisms, followed by Candida spp (predominantly *Candida auris)*, accounting for 27% of CRBSI cases.

**Conclusion:**

Our study showed poor sensitivity and low positive predictive value of catheter tip cultures in diagnosing CRBSI. Employing catheter-sparing methods, such as evaluation of differential time to positivity and quantitative paired blood cultures, will improve the diagnosis of CRBSI, and can lower hospital expenses.

**Disclosures:**

**All Authors**: No reported disclosures

